# Toxicity of the Herbicide Roundup Original DI^®^ in Tadpoles of *Physalaemus erikae* and *Physalaemus cicada* (Anura: Leptodactylidae)

**DOI:** 10.3390/toxics13010004

**Published:** 2024-12-25

**Authors:** Mario Barbosa da Silva Junior, Renan Nunes Costa, Iuri Ribeiro Dias, Yvonnick Le Pendu, Mirco Solé, Caio Vinícius de Mira-Mendes, Victor Goyannes Dill Orrico

**Affiliations:** 1 Tropical Herpetology Lab, Graduate Program in Zoology, Department of Biological Sciences, State University of Santa Cruz, Ilhéus 45662-900, BA, Brazil; irdias@uesc.br (I.R.D.); mksole@uesc.br (M.S.); 2 Department of Biological Sciences, State University of Minas Gerais, Carangola 36800-000, MG, Brazil; renan.costa@uemg.br; 3 Department of Biological Sciences, State University of Santa Cruz, Ilhéus 45662-900, BA, Brazil; yvonnick@uesc.br; 4 Herpetology Section, Zoologisches Forschungsmuseum Alexander Koenig, D-53113 Bonn, Germany; 5 Department of Biology, State University of Maranhão, São Luís 65055-310, MA, Brazil; caiomendes@professor.uema.br

**Keywords:** survival, LC50, acute exposure experience, glyphosate, chronic exposure experience

## Abstract

Brazil is one of the largest consumers of herbicides in the world, and glyphosate-based herbicides (e.g., Roundup^®^) are commonly applied in cropland. Among vertebrates, amphibians are especially susceptible to contamination due to their strong association with freshwater environments. However, our knowledge of how these herbicides affect amphibians is still scarce, mainly regarding the impacts of commercial formulations. In this study, we experimentally evaluated the effects of Roundup Original DI^®^, a glyphosate-based herbicide, on tadpoles of *Physalaemus cicada* and *Physalaemus erikae*. Spawnings were collected in south and southern Bahia, transported to the laboratory, and kept until the tadpoles reached developmental stage 25. Tadpoles were acclimated for 24 h and subsequently exposed to increasing herbicide concentrations at acute and chronic levels to assess survival, lethal concentration (LC50 96 h), and morphological and ontogenetic responses. Contamination significantly decreased the survival of tadpoles of both species and affected the development of tadpoles of *P. erikae.* The estimated lethal concentration (LC50) after 96 h for *Physalaemus cicada* was 5.52 mg a.i./L, and *Physalaemus erikae* was 3.40 mg a.i./L. Also, at chronic concentrations, morphological changes were observed in the tadpoles of *P. erikae*, with the changes being mainly in the tadpoles’ tail and body shapes. Therefore, Roundup Original DI^®^ is considered moderately toxic to both species. Our results extend the knowledge regarding the action of pesticides mainly for species of the genus *Physalaemus*, which is the best-known genus for glyphosate based-herbicide toxicity.

## 1. Introduction

Brazilian biomes are being replaced by extensive monocultures to ensure the production and export of commodities, such as soybeans and sugarcane [1]. A large production of high-quality products without the negative effects of agricultural pests is necessary to maintain this economic export system. For this purpose, farmers apply tons of pesticides, thus contributing to environmental contamination and health problems [1,2,3,4].

The intensive use of pesticides in agriculture has been discussed by society in general, either due to public health problems or environmental impacts resulting from the contamination of biological communities and ecosystems. One of the most discussed and studied environmental effects is the possible action of these pesticides on non-target species since several of the pesticides are non-selective [5,6,7,8,9]. Glyphosate-based herbicide formulations (e.g., Roundup) are highly applied non-selective pesticides in Brazilian crops, with a volume commercialized significantly higher than that of other active ingredients [10]. Its sales for the year 2018, for example, (about 195 kilotons) were twice as high as the second best-selling active ingredient in the country, being applied in most types of agricultural land [1,2,10].

The amount and frequency of application added to the high proximity between bodies of water and agricultural land [11] can lead to contamination of freshwater environments [1,12,13]. Glyphosate has already been detected in surface waters at concentrations of 144 μg/L, 700 μg/L, and 1000 μg/L [14,15], exceeding the limit established by Brazilian legislation (i.e., CONAMA Resolution 357/2005 [16], which determines the maximum permissible amount of 280 μg/L of glyphosate for class III surface waters). Class III waters represent bodies of water intended for human consumption, after conventional or advanced treatment, irrigation of tree crops, cereals and forages, amateur fishing, secondary contact recreation, and the desiccation of animals. 

Amphibians are strongly associated with freshwater environments and are especially susceptible to contamination due to their permeable skin [17]. Therefore, amphibians are good bioindicators, especially of water quality, because, in addition to being found in various types of bodies of water, they have a wide geographical distribution [18,19,20,21,22]. Many species colonize temporary and permanent ponds in agricultural landscapes, with aquatic eggs and tadpoles developing under pesticide stress [17,23,24,25,26]. The larval form of anurans is widely used in ecotoxicological tests since this form can be studied under laboratory conditions, allowing us to verify the possible effects of pesticides in the natural environment [27,28,29,30,31].

Experimental studies with tadpoles have found that commercial glyphosate formulations negatively affect different species of amphibians, decreasing their survival through changes in behavior, anomalies, and morphological malformations [27,30,32,33,34,35]. Some Brazilian species were experimentally tested, but there is a significant gap in ecotoxicological studies when compared to the high diversity of existing amphibians [36]. The number of species in which the sublethal effects of glyphosate were evaluated in amphibians is much lower compared to studies of lethal effects, with few studies that verified the effects on the development of tadpoles [37,38,39,40,41,42] and some that evaluated its effects on the morphology and behavior of tadpoles [27,29,33,35,41]. Also, few similar species have been tested, reducing the potential for comparison between them.

In Brazil, the genus *Physalaemus* (Fitzinger 1826) is the most extensively studied for the lethal effects of glyphosate formulations on tadpoles. However, of the 49 species of the genus, there are studies only for *Physalaemus albonotatus* [43], *Physalaemus cuvieri* [27,44], *Physalaemus centralis* [33], and *Phylsalaemus gracilis* [44]. New studies with species of this genus, in addition to giving more substance to interspecific comparisons, as suggested by some authors [25,34,43], also allow us to learn more about glyphosate formulations and their effects on biodiversity and, consequently, give us the ability to use the results as mitigating measures, which may thus favor other species.

Thus, the objective of this study was to evaluate the lethal and sublethal effects of contamination by a glyphosate formulation Roundup Original DI^®^ on tadpoles of *Physalaemus cicada* Bokermann 1966 and *Physalaemus erikae* Cruz and Pimenta 2004. We aimed to (i) evaluate the survival of tadpoles of the two species exposed to increasing concentrations of the contaminant, (ii) define a lethal concentration for half of a tadpole population (LC50) for both species, and (iii) assess the effects of chronic exposure to the contaminant on the development and morphology of *P. erikae* tadpoles.

## 2. Materials and Methods

### 2.1. Collection and Handling of Spawning

We collected four spawns of *P. erikae* in the municipality of Uruçuca (14°36′35.5″ S; 39°21′24.3″ W) and five spawns of *P. cicada* in the municipality of Brumado (14°3′53.26″ S; 41°51′0.89″ W), both in the state of Bahia, Brazil. The spawns were collected manually with the aid of sieves and packed in plastic bags with water from the pond for transport. These were taken to the Tropical Herpetology Laboratory at the State University of Santa Cruz (UESC, Ilhéus, Bahia, Brazil) where they were placed in glass containers with dechlorinated water, at a temperature of 25 °C so that tadpoles could develop until stage 25.

### 2.2. Experimental Procedure

#### 2.2.1. Acute Experiment (*Physalaemus cicada* and *P. erikae*)

Two experiments were carried out separately, one for *P. cicada* and another for *P. erikae*. The experiments started with tadpoles at stage 25 in both species [45]. We tested the Roundup Original DI^®^—Monsanto, a glyphosate-based herbicide with 44.5% of active ingredient. It is important to highlight that the commercial formulation represents a mixture of glyphosate with other substances, which can increase the toxicity of the active ingredient. However, we calculated the tested concentrations based on the nominal values of glyphosate present in the formula, which does not exclude potential effects associated with the action of other substances in the formulation Roundup Original DI. Five treatments with the following increasing glyphosate concentrations were applied: Control = 0 mg a.i./L; T1= 0.28 mg a.i./L; T2 = 1.5 mg a.i./L; T3 = 3 mg a.i./L; T4 = 6 mg a.i./L. To achieve these glyphosate concentrations, we added 0.63 µL (T1), 3.37 µL (T2), 6.74 µL (T3), and 13.48 µL (T4) of Roundup Original DI^®^. The concentration of T1 was defined based on CONAMA 357 resolution, as this is the maximum concentration allowed by this resolution. The other concentrations were based on recent work regarding glyphosate contamination effects [27,29,43,46]. Experiments had a single application of the contaminant within the period evaluated.

The experiment with *P. cicada* had 10 tadpoles randomly distributed per aquarium. Each treatment had a number of 7 replicates, resulting in a total of 35 experimental units and 350 tadpoles used. Plastic aquariums (27 cm × 18 cm × 8 cm) were used with 1 L of dechlorinated water, without substrate, and with constant oxygenation through aquarium air compressors. For the experiment with *P. erikae*, 10 tadpoles were distributed per aquarium, with a number of 6 replicates per treatment, totaling 300 tadpoles of *P. erikae*. The experiment with *P. erikae* used 30 glass aquariums (20 cm × 15 cm × 10 cm) with 1 L of dechlorinated water, without substrate and with constant oxygenation through aquarium air compressors.

For both experiments, the position of the experimental units and treatments were randomized. The tadpoles were kept in the aquariums for 24 h for acclimatization before applying the herbicide. Tadpoles were exposed to the herbicide for 96 h, and during this period, no feeding was provided. Every 24 h, the number of individuals killed by aquarium was recorded. These were removed and preserved in 10% formalin; during this period, we also checked the temperature and pH of the water.

#### 2.2.2. Chronic Experiment (*Physalaemus erikae*)

The chronic experiment was carried out only for *P. erikae*. Tadpoles were submitted to control treatment with 0 mg a.i./L and a treatment (T) with 2 mg a.i./L; concentration lower than LC50-96 h estimated in the acute experiment.

Each treatment had 10 replicates with six tadpoles randomly distributed per aquarium, totaling 20 experimental units and 120 tadpoles used. The tadpoles were kept for 24 h for acclimatization; after this period, the herbicide was added to the aquariums of the treatment. Every 24 h, we verified the occurrence of dead individuals in the experimental units and also checked the temperature for constancy. Tadpoles were fed *ad libitum* with flocculated feed for ornamental fish (Alcon Basic). The aquariums had constant oxygenation through aquarium air compressors. The experiment had a total duration of 12 days of exposure of the tadpoles to the herbicide. The water of all experimental units was renewed in an interval between five days. We then renewed contamination in the experimental units of the treatment to ensure a similar level of contamination during the days of exposure.

At the end of the experiment, the surviving tadpoles were euthanized with immersion in benzocaine hydrochloride solution (in 1 mg/g solution). After death, the specimens were fixed in 10% formalin. This method followed the protocol determined by Ordinance N°. 148/2012 of the Federal Council of Biology and the protocols approved by Normative Resolution, N°. 12. 20/09/2013 of the National Council for Animal Experimentation Control (CONCEA) and was certificated by the University’s Animal Ethics Committee (CEUA 026/18). Next, photographs of tadpoles were taken for morphological analysis. For this, we used NIKON’s COOLPIX W300 camera (Nikon, Bonn, Germany), supported with a tripod at a distance of five centimeters from the tadpoles, positioned in a Petri dish with ultrasound gel and water. The developmental stage was also evaluated [45], serving as a response variable together with the morphological analysis.

##### Geometric Morphometry (*Physalaemus erikae*)

A total of 100 tadpoles were used to be examined for shape, 50 of which were control and 50 were of treatment. Tadpoles varied between stages 25 and 37 [45]. Eleven landmarks were defined in dorsal view and 22 in lateral view [47,48] (Figure S1). Landmarks were processed using the TPS Dig2 software version 2.31.

##### Linear Morphometry (*Physalaemus erikae*)

Measured morphological traits were total length, body length, tail length, height of caudal musculature, maximum tail height, dorsal fin height, ventral fin height, interocular distance; tail muscle width; body width, (Figure S2) [30,49,50]. The measurements were made from photos, through the ImageJ software (Version 1.54j), of a total of 100 photos, 50 controls, and 50 treatments.

### 2.3. Data Analysis

In the acute experiment, we used the survival rate at the end of exposure as the response variable and performed a Kruskal–Wallis test to compare the survival rate between the different treatments. Next, a Mann–Whitney test with Bonferroni correction was performed for post hoc comparison. To calculate the LC50-96 h, we used Probit regression analyses in the R software (Version 4.4.1) with the ecotox package.

To evaluate shape changes, we first applied the Procrustes Superposition method to remove the effects of size, position, or rotation on shape variation [51]. We also generated a covariance matrix to perform morphometric analyses. Next, we performed a Discriminant Function Analysis (DFA) to generate the final variations between control and treatment. We also performed a Permutation test (10,000 randomizations) to evaluate a statistical significance in the shape difference between treatments. All these analyses were performed in Morphoj software, version 1.07a [52].

We adjusted the linear morphological measurements by dividing them by the total length of the individuals, as larger animals generally have larger measurements. We used the division values as estimates of the variables independent of size. To verify the normality of the data, we conducted the Shapiro–Wilk test, which revealed that the morphological variables did not exhibit a normal distribution. Consequently, we employed the Wilcoxon test to determine whether exposure to the herbicide had a significant impact on the size (growth) and development of tadpoles in each treatment. Additionally, we performed a multivariate analysis using the Hotelling T² test to compare the treatment group with the control.

## 3. Results

### 3.1. Acute Experiment

**Figure 1:**
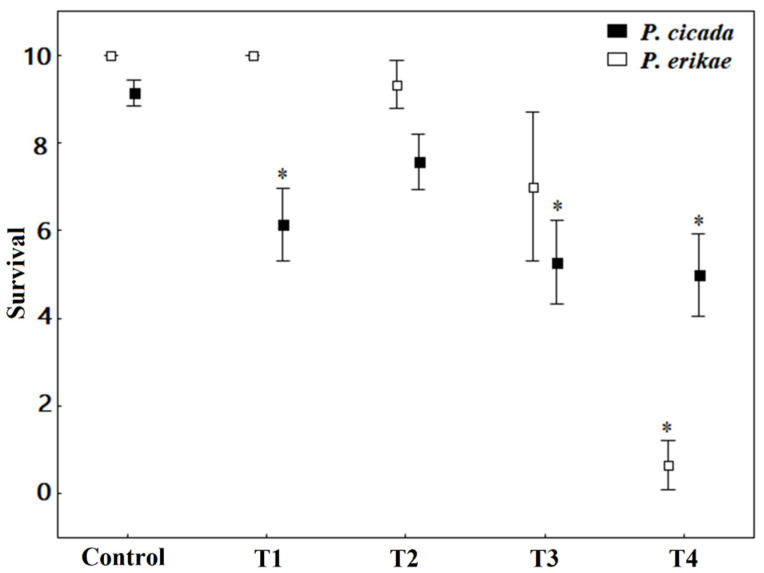
Number of surviving tadpoles of *Physalaemus cicada* and *Physalaemus erikae* in each concentration of Roundup Original DI^®^ at the end of the acute experiment. The squares represent the averages in each treatment and the bars the ± 95% confidence interval. The statistical differences in relation to the control are marked with an asterisk (*), based on the Mann–Whitney test.

**Figure 2:**
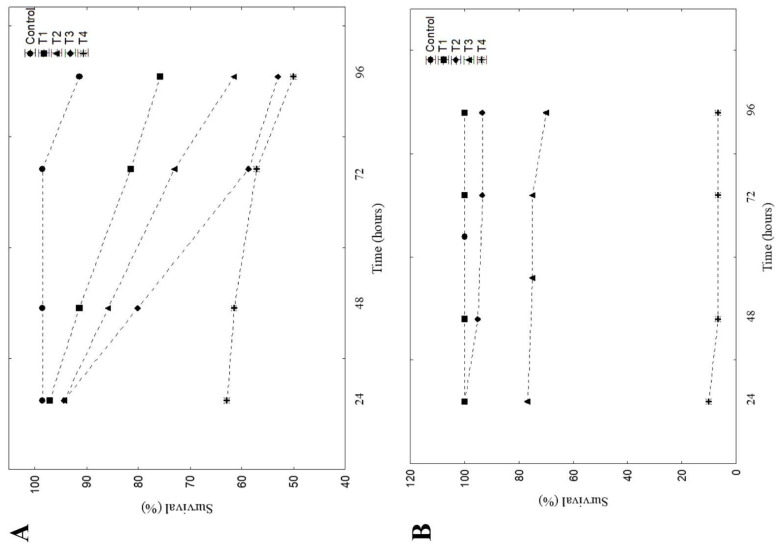
Survival graph over time of tadpoles of *Physalaemus cicada* (**A**) and *Physalaemus erikae* (**B**) submitted to acute concentrations of the Roundup Original DI^®^.

Roundup Original DI^®^ contamination decreased *P. cicada* tadpole survival (H (4) = 17.501, *p* = 0.001—Figure 1). When compared to the control, the lowest survival was observed in T4 (50% mortality—*p* = 0.004), T3 (47.2% mortality—*p* = 0.009), T1 (38.6% mortality—*p* = 0.042), and T2 (24.3%—not significant), respectively. The lethal concentration (LC50 96 h) of *P. cicada* was 5.52 mg a.i./L. For *P. erikae*, the control group and T1 did not present tadpole mortalities at the end of the experiment. Exposure to other concentrations of Roundup Original DI^®^ also decreased the survival of tadpoles of *P. erikae* (H (4) = 21.23, *p* < 0.001—Figure 1). Compared to the control, the highest mortality at the end of the experiment was observed in T4 (93% mortality, *p* = 0.003), T3 (30% mortality, *p* = 0.054), and T2 (7% mortality, *p* = 0.336). The lethal concentration (LC50 96 h) of *P. erikae* was 3.40 mg a.i./L. The survival graph over time can be seen in Figure 2 for both species.

### 3.2. Chronic Experiment (Physalaemus erikae) 

#### 3.2.1. Development

A total of five tadpoles died during the chronic experiment, four in control and one in treatment. When comparing the stage of development^45^ of the surviving tadpoles in the control and treatment, no significant differences were found (W = 1337, *p* = 0.672), with means of the development stage in control 29.6 ± 3.95 and treatment 29.4 ± 3.97. In addition, the control and T tadpoles also presented a similar distribution for the number of tadpoles in each stage of development. 

#### 3.2.2. Morphology

##### Geometric Morphometry

**Figure 3:**
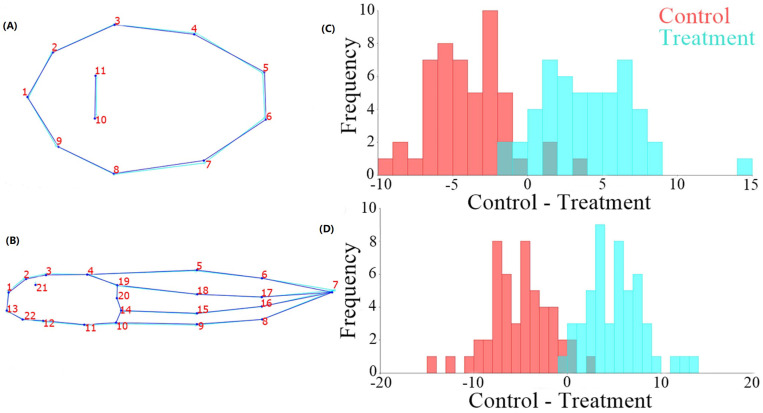
DFA results between control and treatment. (**A**) Graphic reconstructions of the tadpoles’ body in dorsal view (**B**) in lateral view, derived from the deformations obtained with the DFA resulting from the comparison between control (dark blue line) and treatment (light blue line). (**C**) Graph of discriminating score of the difference in shape in dorsal view. (**D**) Graph of discriminating score of the difference in shape in lateral view.

In the DFA between treatment and control, a significant difference was observed in the dorsal plane (Procrustes distance: 0.0225, *p* = <0.01) and lateral plane (Procrustes distance: 0.014, *p* = 0.029). The shape differences are related to the anatomical landmarks of the center of the eye and the point of the end of the tail in the lateral view. In the dorsal view, the differences are in the landmarks on the same transverse line of the posterior edge of the spiracle and at the point of position of the eyes (Figure 3).

##### Linear Morphometry

**Figure 4:**
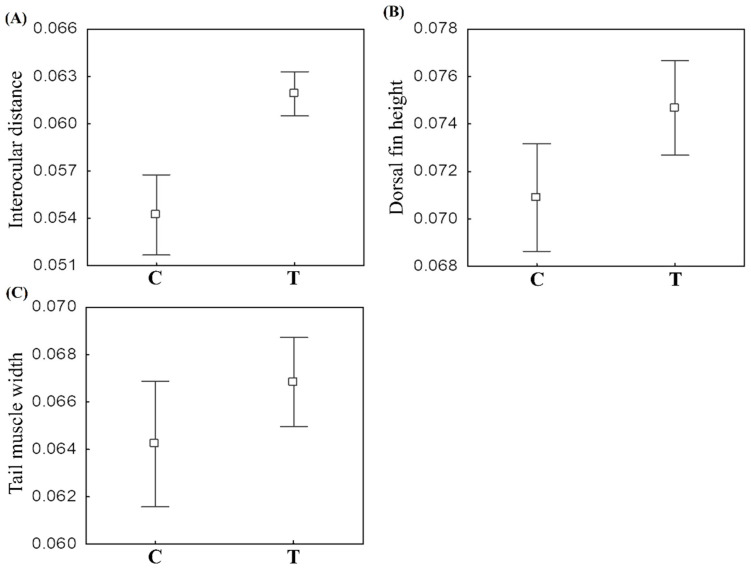
Mean and confidence interval ±95% of (**A**) Interocular distance, (**B**) Dorsal fin height, (**C**) tail muscle width of *Physalaemus erikae* tadpoles in the control and treatment groups at the end of the chronic exposure.

We observed a difference between control and treatment in the linear morphological traits of *P. erikae* (T² = 5.441, *p* < 0.001). Dorsal fin height (W = 913, *p* = 0.020), tail muscle width (W = 021, *p* = 0.023) and interocular distance (W = 346, *p* < 0.001) displayed significant differences between control and treatment (Figure 4).

## 4. Discussion

### 4.1. Acute Experiment

Acute Roundup Original DI^®^ contamination decreased the number of surviving tadpoles of *P. cicada* and *P. erikae*. The absolute difference in acute toxicity values between the two species was 2.12 mg a.i./L, with *P. cicada* being more resistant than *P. erikae* in our sample (LC50-96 h = 5.52 mg a.i./L and 3.4 mg a.i./L, respectively).

The decrease in the survival of tadpoles exposed to glyphosate-based herbicides was also observed for the congeneric species *P. cuvieri* [27], *P. centralis* [33], and *P. albonotatus* [43]. The LC50 estimated after 96 h of *P. cicada* is similar to *P. albonotatus* [43], approximately twice the value found for *P. cuvieri* [27] and four times lower than that found for *P. centralis* [33]. The LC50-96 h of *P. erikae* was the second-lowest found for the genus, above only *P. cuvieri* [27] and almost six times lower than *P. centralis* [33] and 36% lower than *P. albonotatus* [43].

Studies that evaluated tadpoles of the genus Physalaemus contaminated by commercial formulations based on glyphosate and also by pure glyphosate exhibited a range of LC50-96 h values, spanning from 2.13 to 19.7 mg a.i./L. These findings highlight significant variations in toxicity within species of the same genus, a variation that may be further enhanced by the diverse formulations of pesticides that present surfactant substances, like aminomethylphosphonic acid (AMPA) or polyoxyethylene tallow amine (POEA), which may or may not increase toxicity [44,53,54,55]. Also, these differences in species-specific tolerance can be largely explained by the methodological differences of the studies (Table 1). Some pesticides in the commercial formulation have much higher toxicity than the active ingredient of the product itself due to these formulations presenting surfactants that may have higher toxicity than the active ingredient [39,42,46,56,57,58,59,60], which highlights the need for further studies with different formulations.

There are several ecotoxicological studies with tadpoles in literature, but the power of comparisons between species seems uncertain and limited. We can list two main causes: (i) few studies with closely related species and (ii) differences in protocols and methodological conditions. Greater differences in experimental conditions (e.g., differences in commercial formulations, exposure time, number of repetitions, experimental conditions, stage/age of organisms, and others) (e.g., Table 1) in addition to greater phylogenetic distances between analyzed species make it difficult to compare and identify which clades or species are most sensitive [34,43,44]. However, even studies among similar species are subject to variables that hinder comparisons. A good example is studies using species of *Physalaemus* genus. Studies conducted with the species *P. cuvieri* [27], *P. centralis* [42], *P. albonotatus* [43], and the present study (*P. cicada* and *P. erikae*) are methodologically similar. However, there are differences in the commercial formulation tested, which present different types of “inert ingredients”, with different concentrations of surfactants (e.g., POEA and AMPA). The present study uses Roundup Original DI^®^ with 44.5% active ingredient, while *P. cuvieri* was tested using Roundup Original ^®^ with 48% [27]. *Physalaemus centralis* was tested using Glyphosate 480 Agripec^®^ with 48% [42], and *P. albonotatus* with Gliz^®^ 480 SL with 48% [43].

Another complicating factor is the stress level of the spawning environment. Some populations may be more tolerant of pesticides than others because they live within and/or closer to agricultural areas that suffer a periodic application of pesticides [23,24,61,62]. This is the case of *P. centralis* [42], the species of *Physalaemus* genus that displayed greater tolerance to glyphosate formulation tested. This tolerance can be explained by the spawning collection area used in the experiment since the spawns used in the experiment were collected at distances of 10 to 50 m from plantations under pesticide periodic application [42]. Another complicating factor is the low number of individuals used in the experiments. Experiments with *P. centralis* and *P. albonotatus* [42,43] used only 20 individuals in total, different from the most recent studies that used 200 to 500 individuals in experiments to determine LC50 [27,56,62,63]. Despite methodological differences, some authors suggest that the discrepancy between LC50 values may represent species-specific tolerance [44,64,65].

The geographical distribution of *Physalaemus* species included in this comparison covers most of Brazil and represents different morphoclimatic domains that are highly suppressed by agricultural land where pesticides are used. *Physalaemus cicada* is found in most of northeastern Brazil; *P. erikae* is found in the southern region of the state of Bahia in the Atlantic Forest area; *P. centralis* and *P. albonotatus* are found in the southeast and midwest, while *P. cuvieri* has occurrence in almost all regions of the country [66]. This widely distributed group of species can be used as a bioindicator tool [67,68]. An increase in knowledge about widely distributed specific groups, such as the genus *Physalaemus,* can help develop mitigation strategies to reduce the impacts of glyphosate-based herbicides.

### 4.2. Chronic Experiment

The sublethal concentrations used in our study were intended to cause effects on the development of tadpoles, and during the whole experiment, only one tadpole died in the treatment, so the Roundup Original DI was not acutely toxic in this experiment. Although the chronic exposure of *P. erikae* tadpoles to formulation did not have an effect on their development, morphological changes were observed in tadpoles exposed to treatment when compared to control tadpoles.

Some studies have evaluated the effect of glyphosate-based herbicides on amphibian development [37,38,39,40,41,42]. While some noticed that chronic exposure can either prolong the larval period or accelerate metamorphosis [37,39,42], others have observed the absence of effects on the metamorphosis of tadpoles of some species [38,40,41]. All these studies lasted between 30 and 60 days since they carried out experiments until the metamorphosis of tadpoles, unlike ours, which lasted for 12 days of exposure to the herbicide. In the present study, in addition to evaluating the effect on development, we also evaluated the effect on tadpole morphology. Therefore, when the first tadpoles began to reach stage 39, we finished the experiment, since from stage 40 the total length of the tadpole began to decrease due to the resorption of the tail [45]. However, the 12 days of exposure to the herbicide were enough to produce effects.

Although there is no difference in the development between control and treatment tadpoles, we verified the morphological effects of contamination by Roundup Original DI^®^. Our results showed that the morphology of *P. erikae* tadpoles is significantly affected by sublethal glyphosate concentrations in commercial formulation. This caused changes in the shape of the tadpoles’ bodies in addition to changes in morphological traits, such as dorsal fin height, tail muscle width, and interocular distance. These changes in the morphology of *P. erikae* tadpoles caused by glyphosate exposure may have several implications for the life of these animals, both in the larval and adult phases.

One of the most visible changes in the shape of the tadpoles’ body was in the width of the body in the position of the spiracle in dorsal view. The control tadpoles showed the reference points of this position more distant, and this can be interpreted as a wider body when compared to the treatment tadpoles [69]. We also recorded in tadpoles exposed to Roundup Original DI an increase in the height of the dorsal fin, along with an increase in the width of the tail musculature, contrary to our expectations (we expected a decrease in these structures). A change in the shape of the tadpole tail was also observed, where it is possible to observe a displacement at the tip of the tail when comparing the tadpoles of control and treatment. As has already been suggested by some authors, changes in these characteristics mainly influence speed and maneuverability during tadpole swimming, impacting their survival [69,70,71,72].

Within the genus *Physalaemus,* sublethal effects of glyphosate on the development of tadpoles were evaluated only in *P. cuvieri* and *P. centralis* [27,28,29,30,31,32,33]. They observed that sublethal concentrations of glyphosate-based herbicides in tadpoles of *P. centralis* increase the body size of shapeshifters while Picloram, depending on the concentration, can cause an increase or decrease in the body [33]. For the tadpoles of *P. cuvieri* exposed to Roundup Original, a greater fluctuating asymmetry was observed in the nostril–snout distance and in the width of the eyes [27]. This shows that sublethal effects of pesticides on amphibians can vary widely.

Changes in the morphology of tadpoles exposed to commercial formulations of glyphosate-based herbicides can imply the success or failure of these animals in various activities, such as getting food and fleeing predators. The effects of these morphological changes are still uncertain. What we do know is that exposure to pesticides can affect tadpoles in various ways and can cause anomalies and malformations [73,74,75], in addition to increasing or decreasing the size of tadpoles and important structures for the fitness of these animals. All these morphological changes demonstrate the need for further studies with this approach, especially when we take into account that glyphosate can persist in the environment between 76 and 240 days [76,77], so amphibians are exposed to these pesticides for an even longer period.

In natural environments, glyphosate concentrations (as well as inert ingredients in the commercial formulations) vary according to land use and the structural characteristics of water bodies (e.g., depth, water speed, presence of submerged vegetation, soil characteristics), which may or may not facilitate the accumulation of the herbicide [78,79]. Lentic environments (e.g., reservoirs, permanent and temporary ponds) are more likely to have higher concentrations of glyphosate. These environments are frequently used by various species of amphibians, such as those of the genus Physalaemus, commonly found breeding in temporary ponds in agricultural lands. Therefore, for a more accurate risk assessment, it is important to evaluate the concentrations of glyphosate found in the reproductive environments of amphibians. Brazil ranks among the largest consumers of glyphosate-based pesticides globally [80]. Glyphosate and its metabolite AMPA have been detected in the majority of water samples analyzed by researchers, often exceeding levels stipulated by Brazilian regulations [80,81,82]. Instances of glyphosate and/or AMPA contamination in freshwater environments worldwide, such as streams, lakes, and wetlands, range from 0.75 to 7.6 mg a.i./L, e.g., Giesy [58,80,83,83,84,85,86,87], with numerous contamination reports surpassing detectable limits by current methodologies [82]. Therefore, we posit that the contamination levels simulated in this study realistically represent the presence of Roundup Original DI in aquatic environments, comparable to concentrations commonly employed in toxicity bioassays conducted with tadpoles.

## 5. Conclusions

The results of this study show that the Roundup Original DI^®^, a glyphosate-based herbicide, has a major impact on amphibian species tested. The mortality of the tadpoles of *P. cicada* and *P. erikae* in this study places glyphosate as moderately toxic to these species [36,58]. Comparisons in relation to other studies should be made with caution, especially when the experimental protocols present great methodological differences.

Our results also showed that *P. erikae* larvae exposed to sublethal concentrations of Roundup Original DI^®^, develop differences in their morphology compared to larvae without herbicide exposure. These differences can have several influences on the survival of these animals, as they can interfere with the ability to feed, reproduce, or escape a predator.

We conclude that, although the genus *Physalaemus* is one of the better-studied groups regarding the toxicological effects of pesticides, there is still a large gap to having a real understanding of how these species are being affected. There is also a need for methodological structuring of how to evaluate the effects of pesticides on amphibians.

## Figures and Tables

**Table 1:** Methodological differences between studies with similar species that conducted an LC50 toxicity test. N = number; I. = experimental unit; d.s. = development phase.

	*P. albonotatus*	*P. centralis*	*P. cuvieri*	*P. cuvieri*	*P. cuvieri* and *P. gracilis*	*P. erikae*	*P. cicada*
Commercial formulation	Gliz 480 SL	Glyphosate 480 Agripec^®^	Not described	Roundup Original^®^	Roundup Original DI^®^	Roundup Original DI^®^	Roundup Original DI^®^
Glyphosate in the formula	48%	48%	48%	48%	44.5%	44.5%	44.5%
Inert ingredientes	Not described	Not described	684 g.L^−1^ (68.4% m. v-1).	Not described	Not described	Not described	Not described
Treatments	0–0.8–1.6–3.2 –6.4 (mg L^−1^)	0–8–16–32–64–128 (mg/L)	0–3.19–6.36–9.51–12.64–e 15.75 (mg L^−1^)	0–0.38–2–4–6 (mg a.i./L)	0–0.1–0.3–0.4–1.5–2.5–3.5–4.5 (mg a.i./L)	0–0.28–1.5–3–6 (mg a.i./L)	0–0.28–1.5–3–6 (mg a.i./L)
N. of replicates per treatment	4	4	4	9	6	6	7
N. of tadpoles by e.u.	5	5	5	10	5	10	10
N. of tadpoles tested	20	20	120	450	240	300	350
N. of egg masses collected	Three egg masses from different locations, separated by at least 1 km	Six egg masses from different locations, separated by at least 1 km	Not described	Four egg masses from two different ponds, separated by at least 1 km	Not described	Four egg masses in the same pond	Five egg masses in the same pond
D.s. of tadpoles (Gosner 1960)	Not described	25	25	25	25–26	25	25
Description of the collection site	Not described	Temporary ponds located 10–50 meters away from a soybean plantation, where glyphosate has been used for at least a decade	Not described	Two similar ponds with high percentages of Cerrado vegetation on their margins. Low anthropogenic disturbance in the surrounding landscape	One pond in a non-agricultural area of the northern region of the Rio Grande do Sul State, Brazil	Permanent pond, surrounded by an agroforestry system, named “cabruca”, in which cacao grows under the shade of native tree species.	A semi-permanent pond, surrounded by a relatively well-conserved fragment of Caatinga vegetation, without contact with croplands
Tadpoles acclimation in e.u. before exposure	Not described	Not described	Not described	24 h	Not described	24 h	24 h
Site of experiment	Not described	Laboratory	Laboratory	Laboratory	Laboratory	Laboratory	Laboratory
Lab conditions during experiments	Mean temperature was 31.2 °C	Temperature between 28 °C and 32 °C	Not described	Mean temperature 28° ± 2 °C. Controlled photoperiod (12 h light/12 h dark).	Natural photoperiod (natural lighting)	Mean temperature 25 °C ± 1 °C. Controlled photoperiod (12 h light/12 h dark).	Mean temperature 25 °C ± 1 °C. Controlled photoperiod (12 h light/12 h dark).
Experimental units conditions	Plastic aquaria with 1 L of mineral water (mean ph = 4.92)	Circular transparent containers (material not described) with 1 L of rainwater (ph adjusted to 7)	Glass aquaria with 1 L of dechlorinated water	Glass aquaria with 2 L of dechlorinated water, without substrates and constant oxygenation (ph between 7 and 7.2)	Sterile and cylindrical glass vessels of 450 mL capacity.	Glass aquaria with 1 L of dechlorinated water, without substrate and constant oxygenation	Plastic aquaria with 1 L of dechlorinated water, without substrate and constant oxygenation
Exposure time	96 h	96 h	48 and 96 h	96 h	96 h	96 h	96 h
Reference	[43]	[42]	[33]	[27]	[44]	This study	This study

## Data Availability

The data presented in this study are available on request from the corresponding authors.

## Supplementary Materials

The following supporting information can be downloaded at: https://www.mdpi.com/article/10.3390/toxics13010004/s1, Figure S1: Illustrations and descriptions of the location of the reference points of the tadpole morphology in dorsal and lateral view; Figure S2: Linear measurements obtained in lateral view (1); TL (Total length); BL (body length); TAL (tail length); TMH (height of caudal musculature); MTH (Maximum tail height); HD (dorsal fin height); VH (ventral fin height), dorsal view (2); IOD (interocular distance); TMW (Tail muscle width); BW (body width), exemplified in a tadpole of *Physalaemus erikae*.
