# Supplementary material for: Toxicity of the Herbicide Roundup Original DI® in Tadpoles of Physalaemus erikae and Physalaemus cicada (Anura: Leptodactylidae)
Source: Toxics. 2024 Dec 25;13(1):4. doi: 10.3390/toxics13010004 (PMC11768775; doi:10.3390/toxics13010004)

Figure S1: Illustrations and descriptions of the location of the reference points of the tadpole morphology in dorsal and lateral view.

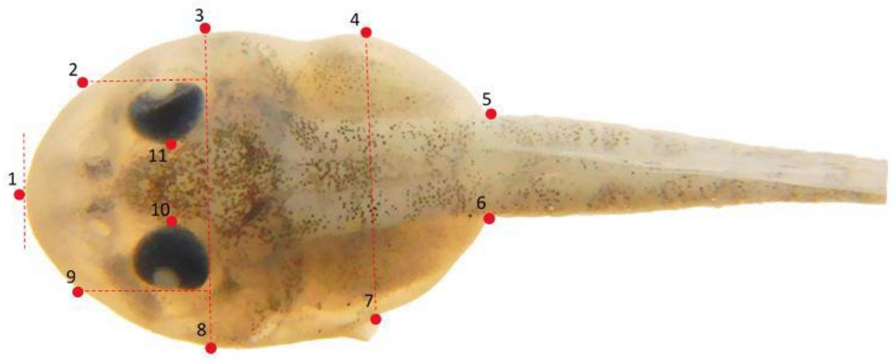

landmarks	Description
1	Tip of the muzzle
2	At the intersection between the anterior edge of the body with a sagittal line that is touching to the more lateral edge of the right eye
3	At the intersection between the right lateral edge of the body and a cross line that is touching the posterior edge of the right eye
4	On the same cross line of the posterior edge of the spirocle
5	Point at the junction of the tail muscle to the right side of the body
6	Point at the junction of the tail muscle to the left side of the body
7	Body edge at the base of the spirocle
8	At the intersection between the left lateral edge of the body and a transverse line that is touching the posterior edge of the left eye
9	At the intersection between the anterior edge of the body with a sagittal line that is touching to the more lateral edge of the left eye
10	Parallel to the inner side of the left eye
11	Parallel to the inner side of the right eye

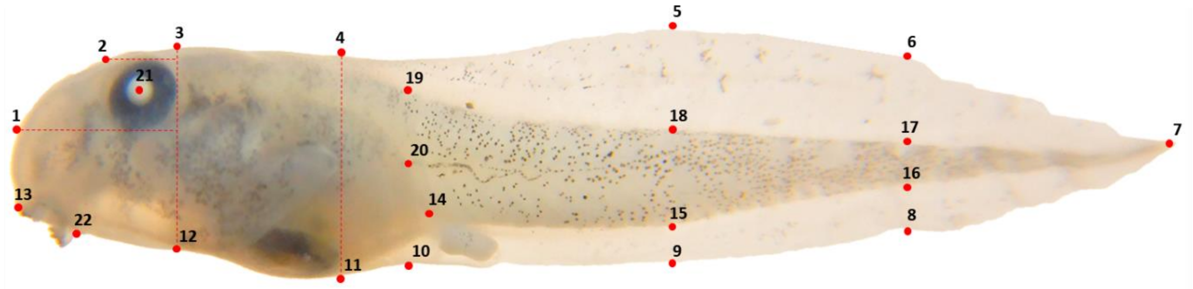

landmarks	References
1	At the intersection between the anterior edge of the body with a sagittal line that is touching to the most basal edge of the eye
2	At the intersection between the anterior edge of the body with a sagittal line that is touching to the upper edge of the eye
3	At the intersection between the dorsal edge of the body with a sagittal line that is touching to the posterior edge of the eye
4	On the same transverse line as the posterior tip of the spirocle
5	Dorsal edge of caudal fin at highest point
6	Dorsal tail border located in the middle between points 5 and 7
7	Tail tip
8	Ventral edge of caudal fin directly below n°. 6
9	Ventral edge of caudal fin directly below n°. 5
10	Ventral edge of the fin directly below n°. 20

11	Ventral edge of the body directly below point 4
12	Ventral edge of the body directly below point 3
13	Anterior point of oral tube when viewed from the side
14	Ventral tail muscle edge at joint with body
15	Ventral edge of the tail muscle directly below point #5
16	Ventral edge of the tail muscle directly below n°. 6
17	Dorsal edge of tail muscle directly below n°. 6
18	Dorsal edge of the tail muscle directly below ventral n°. 5
19	Dorsal edge of the tail musculature directly above point n°. 10
20	Intersection of notochord and body
21	Eye center
22	Posterior point of the oral tube when viewed from the side

Figure S2: Linear measurements obtained in lateral view (1); TL (Total length); BL (body length); TAL (tail length); TMH (height of caudal musculature); MTH (Maximum tail height); HD (dorsal fin height); VH (ventral fin height), dorsal view (2); IOD (interocular distance); TMW (Tail muscle width); BW (body width), exemplified in a tadpole of *Physalaemus erikae* .

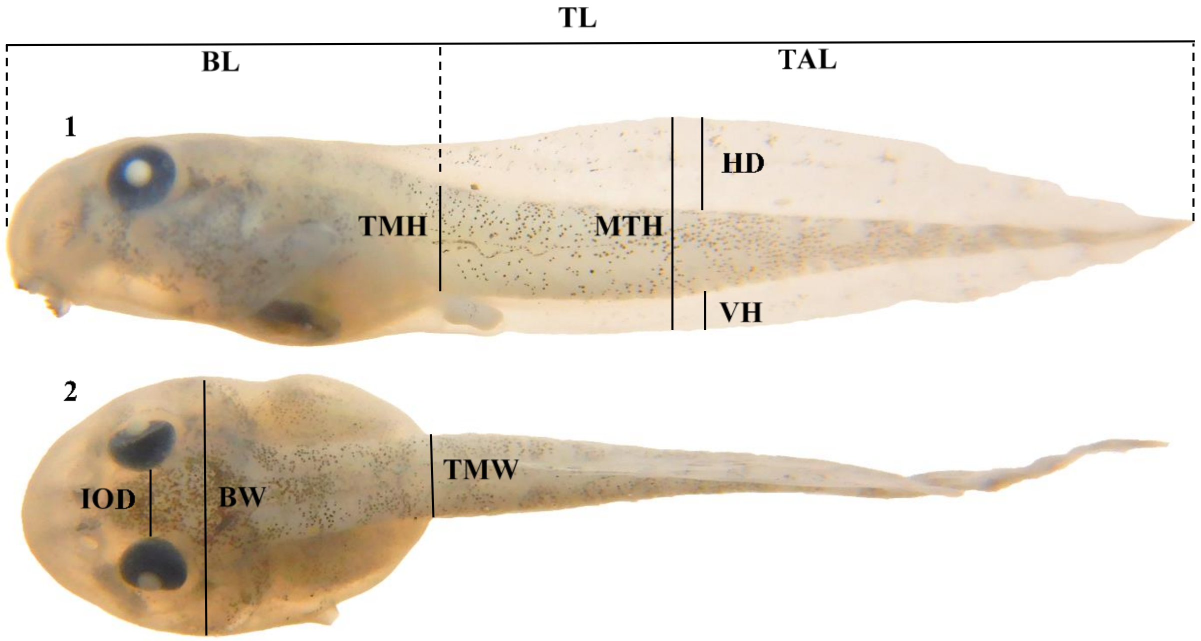

Supplement: Supplementary file 1 [file toxics-13-00004-s001.zip › toxics-3022636-supplementary.pdf]
